# Correction: Probiotics attenuate valproate-induced liver steatosis and oxidative stress in mice

**DOI:** 10.1371/journal.pone.0333517

**Published:** 2025-09-29

**Authors:** Wenfang Song, Xinrui Yan, Yu Zhai, Jing Ren, Ting Wu, Han Guo, Yu Song, Xiaojiao Li, Yingjie Guo

In the Author Contributions, Xiaojiao Li. (XL) should be listed as one of the persons who secured funding for the project that resulted in this publication.

## References

[1] Song W, Yan X, Zhai Y, Ren J, Wu T, Guo H, et al. Probiotics attenuate valproate-induced liver steatosis and oxidative stress in mice. PLoS One. 2023;18(11):e0294363. doi: 10.1371/journal.pone.0294363 37971986 PMC10653412

