# Supplementary figures and images for: Probiotics attenuate valproate-induced liver steatosis and oxidative stress in mice
Source: PLoS One. 2023 Nov 16;18(11):e0294363. doi: 10.1371/journal.pone.0294363 (PMC10653412; doi:10.1371/journal.pone.0294363)

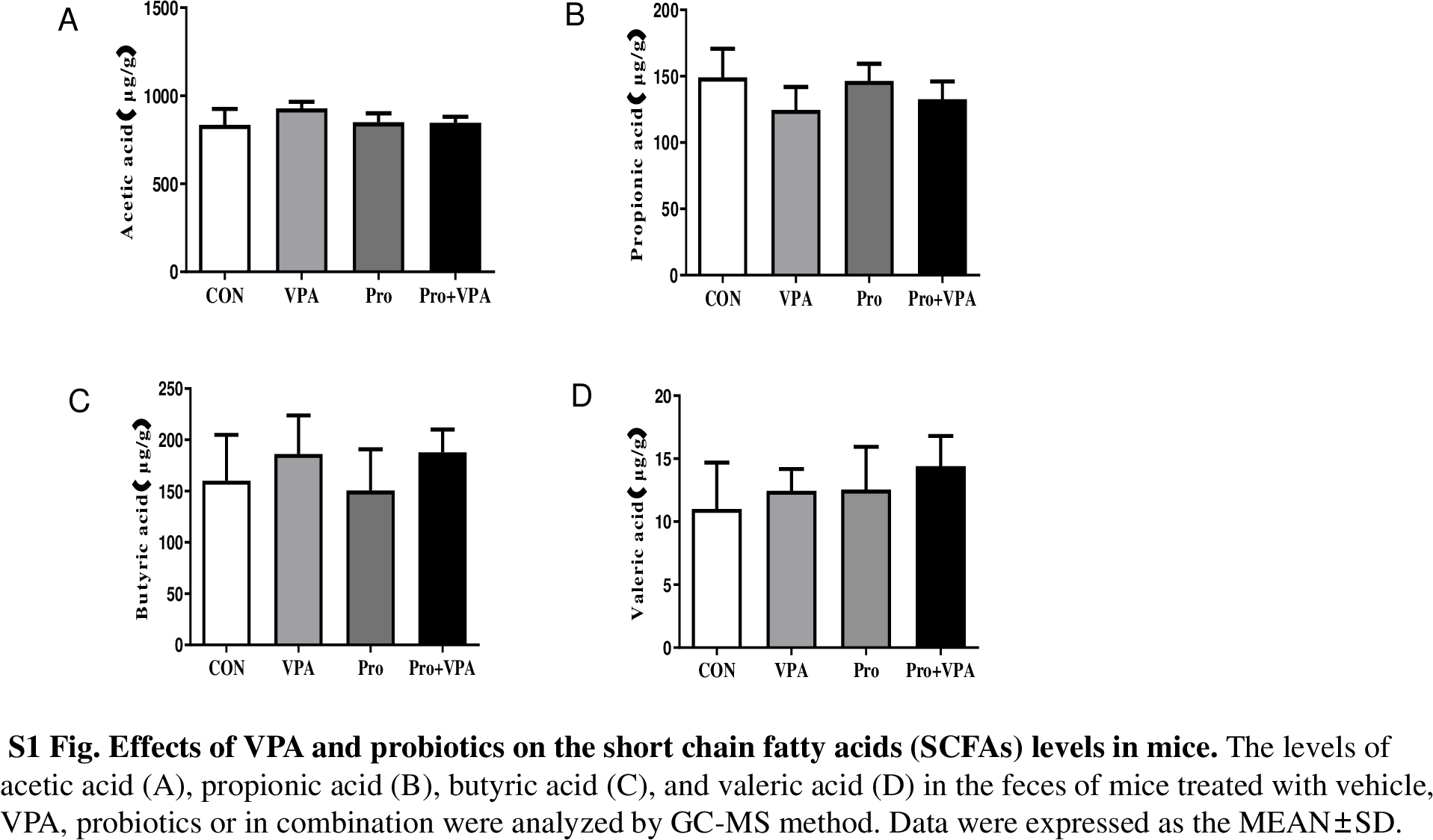

Supplement: S1 Fig — The levels of acetic acid (A), propionic acid (B), butyric acid (C), and valeric acid (D) in the feces of mice treated with vehicle, VPA, probiotics or in combination were analyzed by GC-MS method. Data were expressed as the MEAN±SD. (TIF) [file pone.0294363.s001.tif]

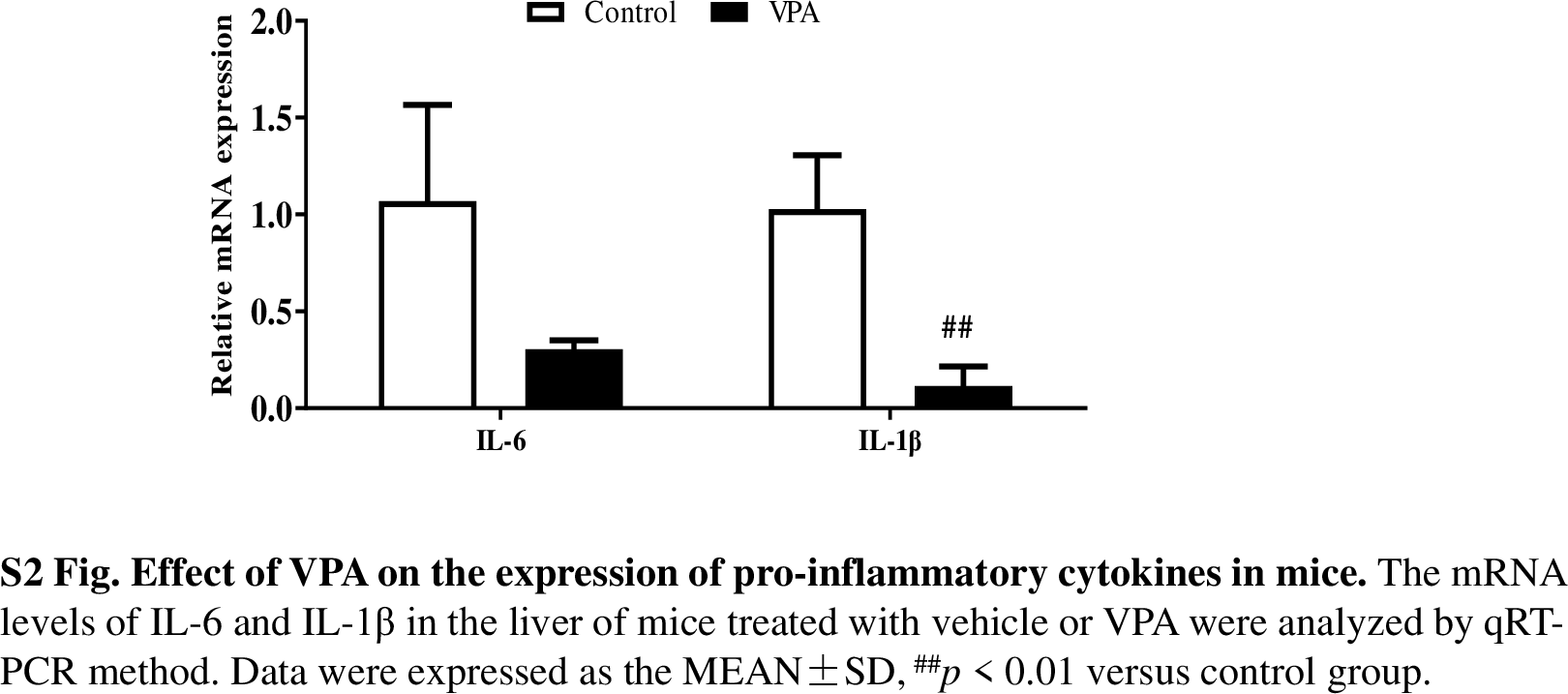

Supplement: S2 Fig — The mRNA levels of IL-6 and IL-1β in the liver of mice treated with vehicle or VPA were analyzed by qRT-PCR method. Data were expressed as the MEAN±SD, ##p < 0.01 versus control group. (TIF) [file pone.0294363.s002.tif]

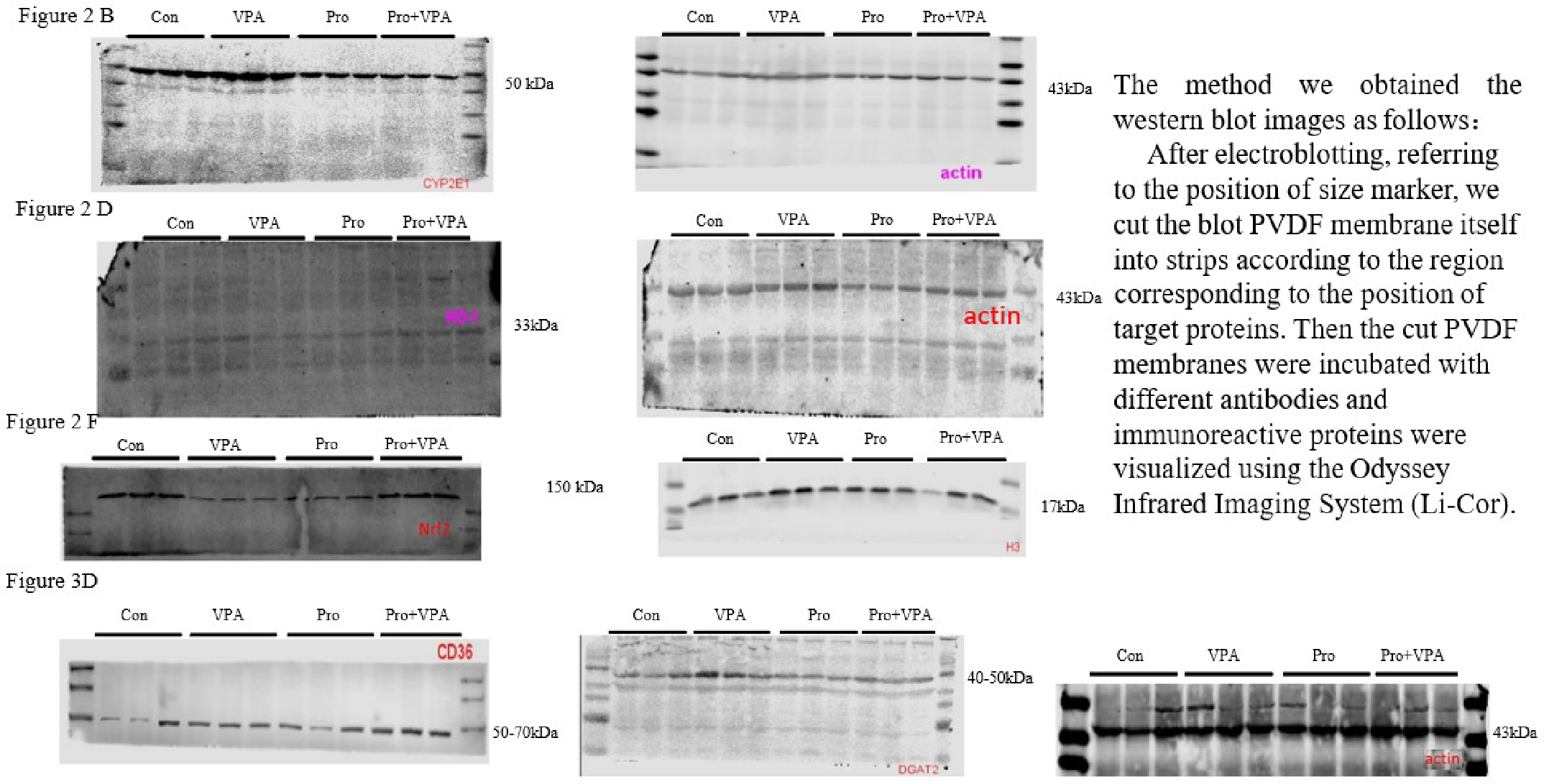

Supplement: S3 Fig — (TIF) [file pone.0294363.s003.tif]

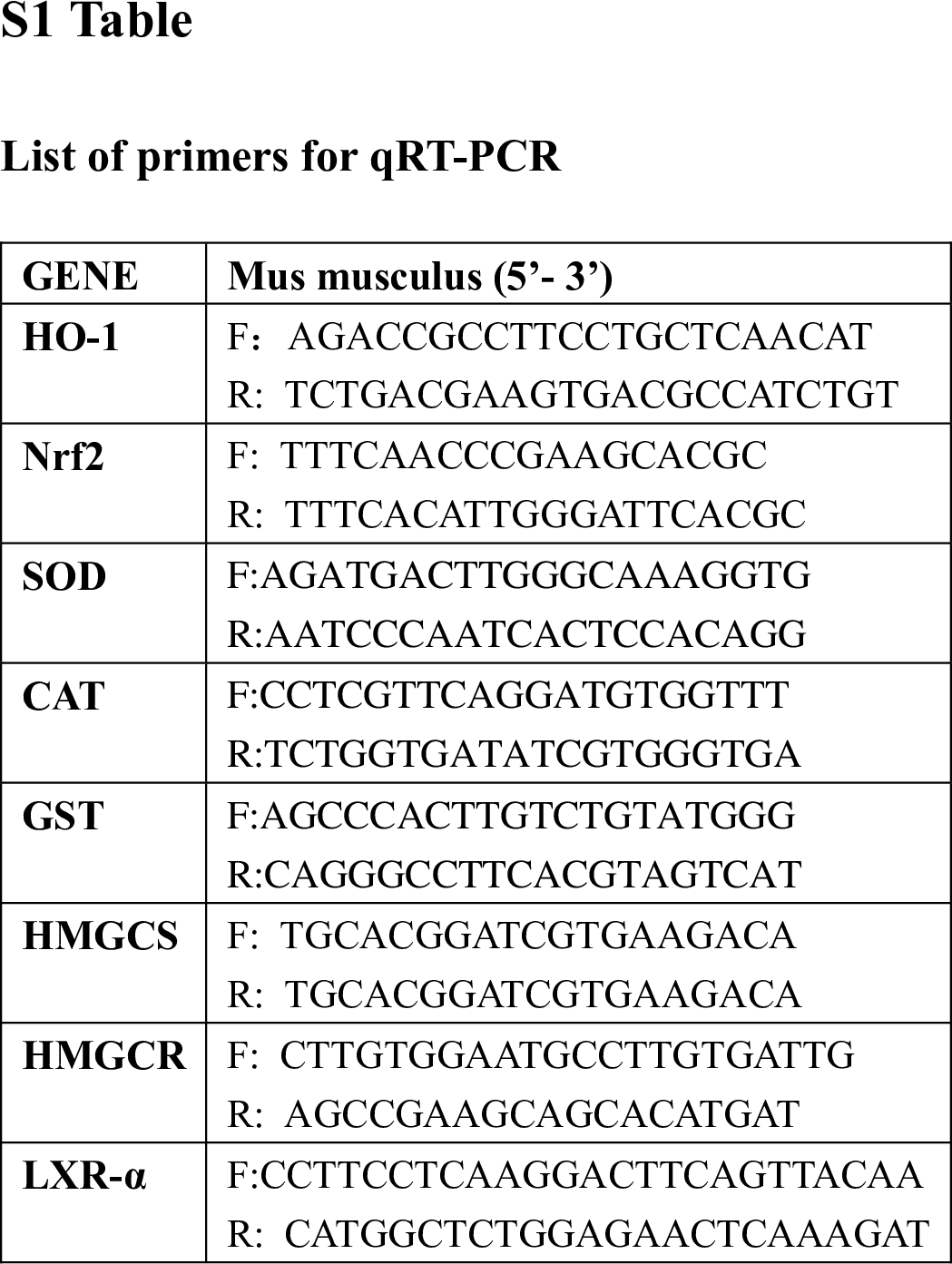

Supplement: S1 Table — (TIF) [file pone.0294363.s004.tif]
