# Supplementary material for: Probiotics attenuate valproate-induced liver steatosis and oxidative stress in mice
Source: PLoS One. 2023 Nov 16;18(11):e0294363. doi: 10.1371/journal.pone.0294363 (PMC10653412; doi:10.1371/journal.pone.0294363)
Supplement: Attachment — Submitted filename: response to reviewers.docx [file pone.0294363.s005.docx]

Dear Dr. Loor，

We sincerely thank you and the reviewers for the helpful comments on our manuscript (PONE-D-23-14625). Those comments are all valuable and very helpful for revising and improving our paper. We have revised the text in order to address the reviewers’ comments. The specific changes have been indicated using the Track Changes feature.

Journal Requirements:

When submitting your revision, we need you to address these additional requirements.

1. Please ensure that your manuscript meets PLOS ONE's style requirements, including those for file naming. The PLOS ONE style templates can be found at 
https://journals.plos.org/plosone/s/file?id=wjVg/PLOSOne_formatting_sample_main_body.pdf and 
https://journals.plos.org/plosone/s/file?id=ba62/PLOSOne_formatting_sample_title_authors_affiliations.pdf

**Response: Thank you for your reminder. We have ensured the manuscriot meets PLOS ONE’s style requierments**.

2. PLOS ONE now requires that authors provide the original uncropped and unadjusted images underlying all blot or gel results reported in a submission’s figures or Supporting Information files. This policy and the journal’s other requirements for blot/gel reporting and figure preparation are described in detail at https://journals.plos.org/plosone/s/figures#loc-blot-and-gel-reporting-requirements and https://journals.plos.org/plosone/s/figures#loc-preparing-figures-from-image-files. When you submit your revised manuscript, please ensure that your figures adhere fully to these guidelines and provide the original underlying images for all blot or gel data reported in your submission. See the following link for instructions on providing the original image data: https://journals.plos.org/plosone/s/figures#loc-original-images-for-blots-and-gels. 
  
In your cover letter, please note whether your blot/gel image data are in Supporting Information or posted at a public data repository, provide the repository URL if relevant, and provide specific details as to which raw blot/gel images, if any, are not available. Email us at [plosone@plos.org](mailto:plosone@plos.org) if you have any questions.

**Response:Thank you for you reminder. We have provided the original underlying images for all immunoblotting data reported in the figure2 B, D and F, and Figure 3C , and uploaded the file named as “S1 Raw images” in the supporting information.**

3. To comply with PLOS ONE submissions requirements, in your Methods section, please provide additional information regarding the experiments involving animals and ensure you have included details on (1) methods of sacrifice, (2) methods of anesthesia and/or analgesia, and (3) efforts to alleviate suffering.

**Response: Thank you for you suggestions. We have provided the information regarding the experiments involving animals (1) methods of sacrifice and (2) methods of anesthesia and/or analgesia,(3) efforts to alleviate suffering in the methods section of revised manuscript.**

4. We suggest you thoroughly copyedit your manuscript for language usage, spelling, and grammar. If you do not know anyone who can help you do this, you may wish to consider employing a professional scientific editing service.

Whilst you may use any professional scientific editing service of your choice, PLOS has partnered with both American Journal Experts (AJE) and Editage to provide discounted services to PLOS authors. Both organizations have experience helping authors meet PLOS guidelines and can provide language editing, translation, manuscript formatting, and figure formatting to ensure your manuscript meets our submission guidelines. To take advantage of our partnership with AJE, visit the AJE website (http://learn.aje.com/plos/) for a 15% discount off AJE services. To take advantage of our partnership with Editage, visit the Editage website (www.editage.com) and enter referral code PLOSEDIT for a 15% discount off Editage services.  If the PLOS editorial team finds any language issues in text that either AJE or Editage has edited, the service provider will re-edit the text for free.

Upon resubmission, please provide the following:
 
The name of the colleague or the details of the professional service that edited your manuscript

A copy of your manuscript showing your changes by either highlighting them or using track changes (uploaded as a *supporting information* file)

A clean copy of the edited manuscript (uploaded as the new *manuscript* file)”

**Response:Thank you for you suggestions. The whole manuscript for language usage, spelling, and grammar using track changes was carefully revised by Shuang Wu, who graduated from School of foreign language, Jinan University. The revised manuscriot with track changes was uploaded as a separate file “Manuscript revised language with track changes” in supporting information.**

5.PLOS requires an ORCID iD for the corresponding author in Editorial Manager on papers submitted after December 6th, 2016. Please ensure that you have an ORCID iD and that it is validated in Editorial Manager. To do this, go to ‘Update my Information’ (in the upper left-hand corner of the main menu), and click on the Fetch/Validate link next to the ORCID field. This will take you to the ORCID site and allow you to create a new iD or authenticate a pre-existing iD in Editorial Manager. Please see the following video for instructions on linking an ORCID iD to your Editorial Manager account: https://www.youtube.com/watch?v=_xcclfuvtxQ

**Response: Thank you for you suggestions. We have ensured the corresponding author’ ORCID iD 0000-0003-4203-8296 validated in Editorial Manager.**

6. We note that you have included the phrase “data not shown” in your manuscript. Unfortunately, this does not meet our data sharing requirements. PLOS does not permit references to inaccessible data. We require that authors provide all relevant data within the paper, Supporting Information files, or in an acceptable, public repository. Please add a citation to support this phrase or upload the data that corresponds with these findings to a stable repository (such as Figshare or Dryad) and provide and URLs, DOIs, or accession numbers that may be used to access these data. Or, if the data are not a core part of the research being presented in your study, we ask that you remove the phrase that refers to these data.

**Response: Thank you for you suggestions. We have added the Figure S1 and Figure S2 in the supporting information, which show the finding of “data not shown” in the discussion section of manuscript. In the revised manuscript, we have substituted the phase “data not shown” with Figure S1 and Figure S2, which are marked with red words of revised manuscript.**

7.Please include your full ethics statement in the ‘Methods’ section of your manuscript file. In your statement, please include the full name of the IRB or ethics committee who approved or waived your study, as well as whether or not you obtained informed written or verbal consent. If consent was waived for your study, please include this information in your statement as well.

**Response: Thank you for you suggestions. We have added the full name of the ethics committee in the Methods section, and all of us obtained informed written consent, as follows “ All animal protocols and experiments were approved by the Animal Ethics Committee of Jilin University, in accordance with the Guidelines for the Care and Use of Laboratory Animals of Jilin University (Protocol Number: 2021-PZ012). Everyone obtained informed written consent”, which were marked with red words in the Methods section of revised manuscript.**

[Note: HTML markup is below. Please do not edit.]

Reviewers' comments:

Reviewer's Responses to Questions

**Comments to the Author**

1. Is the manuscript technically sound, and do the data support the conclusions?

The manuscript must describe a technically sound piece of scientific research with data that supports the conclusions. Experiments must have been conducted rigorously, with appropriate controls, replication, and sample sizes. The conclusions must be drawn appropriately based on the data presented.

Reviewer #1: Yes

2. Has the statistical analysis been performed appropriately and rigorously?

Reviewer #1: No

3. Have the authors made all data underlying the findings in their manuscript fully available?

The [PLOS Data policy](http://www.plosone.org/static/policies.action#sharing) requires authors to make all data underlying the findings described in their manuscript fully available without restriction, with rare exception (please refer to the Data Availability Statement in the manuscript PDF file). The data should be provided as part of the manuscript or its supporting information, or deposited to a public repository. For example, in addition to summary statistics, the data points behind means, medians and variance measures should be available. If there are restrictions on publicly sharing data—e.g. participant privacy or use of data from a third party—those must be specified.

Reviewer #1: Yes

4. Is the manuscript presented in an intelligible fashion and written in standard English?

PLOS ONE does not copyedit accepted manuscripts, so the language in submitted articles must be clear, correct, and unambiguous. Any typographical or grammatical errors should be corrected at revision, so please note any specific errors here.

Reviewer #1: Yes

5. Review Comments to the Author

Please use the space provided to explain your answers to the questions above. You may also include additional comments for the author, including concerns about dual publication, research ethics, or publication ethics. (Please upload your review as an attachment if it exceeds 20,000 characters)

Reviewer #1: The subject is very interesting and useful and the article is very well written.
Please mention the weight of the mice.

**Response: Thank you for you suggestions. We have added the description of the weight of the mice “20-22 g”** **in the section of Animals and treatment of manuscript, which are marked with red words.**

Please depict a graphical abstract including results.

**Response: Thank you for you suggestions. We have added the description of the graphical abstract, as shown below.**

**“ VPA administration to mice enhanced the hepatic oxidative stress and lipid accumulation by increasing the pro-oxidant CYP2E1 level, inhibiting the Nrf2/antioxidant enzyme pathway, and upregulating the CD36 and DGAT2 levels, accompanied with the gut microbiota dysbiosis, all of which were significantly alleviated by co-treatment with probiotics.”**

For statistical analysis of the body weight, repeated measure ANOVA is needed. Please correct it and mention it in the statistical analysis.

**Response: Thank you for your suggestions. For statistical analysis of the body weight, multiple comparision between groups at every time point were performed, but the comparison of body weight within group between different time points was not adopted in the study. Therefore, one way ANOVA with multiple comparison, but not repeated measure was used for the statistical analysis of the body weight in the study. In addition, for clear view of significant differences of the body weight between groups, we have replaced the original line chart with a bar chart, and the body weight were recorded every six days, not every three days, as shown in the revised Figure 1A.**

The experimental study included four groups, and one-way ANOVA is the correct analysis. When do the authors use T student?

**Response: Thank you for your good suggestions. As you described, one-way ANOVA analysis was carried out for all the four groups involved in the experimental results, which have been carefully re-analyzed and shown in revised figures. T-test for comparison between the two groups was involved in Figure 3A. The statistical anaylsis in detail was described in the section of Statistical analysis in the revised manuscript.**

Your Sincerely

Yingjie Guo

School of Life Sciences, Jilin University
