# Supplementary material for: Probiotics attenuate valproate-induced liver steatosis and oxidative stress in mice
Source: PLoS One. 2023 Nov 16;18(11):e0294363. doi: 10.1371/journal.pone.0294363 (PMC10653412; doi:10.1371/journal.pone.0294363)
Supplement: Attachment — Submitted filename: Response to reviewers.docx [file pone.0294363.s006.docx]

Dear Dr. Loor，

We sincerely thank you and the reviewers for the helpful comments on our manuscript (PONE-D-23-14625R1). Those comments are all valuable and very helpful for revising and improving our paper. We have revised the text in order to address the reviewers’ comments. The specific changes have been indicated using the Track Changes feature.

Journal Requirements:

Please review your reference list to ensure that it is complete and correct. If you have cited papers that have been retracted, please include the rationale for doing so in the manuscript text, or remove these references and replace them with relevant current references. Any changes to the reference list should be mentioned in the rebuttal letter that accompanies your revised manuscript. If you need to cite a retracted article, indicate the article’s retracted status in the References list and also include a citation and full reference for the retraction notice.

**Response: Thank you for your reminder. We have ensured that all listed references are complete and correct in the manuscript. Meanwhile, there are not cited papers retracted**

[Note: HTML markup is below. Please do not edit.]

Reviewers' comments:

Reviewer's Responses to Questions

**Comments to the Author**

1. If the authors have adequately addressed your comments raised in a previous round of review and you feel that this manuscript is now acceptable for publication, you may indicate that here to bypass the “Comments to the Author” section, enter your conflict of interest statement in the “Confidential to Editor” section, and submit your "Accept" recommendation.

Reviewer #1: All comments have been addressed

2. Is the manuscript technically sound, and do the data support the conclusions?

The manuscript must describe a technically sound piece of scientific research with data that supports the conclusions. Experiments must have been conducted rigorously, with appropriate controls, replication, and sample sizes. The conclusions must be drawn appropriately based on the data presented.

Reviewer #1: Yes

3. Has the statistical analysis been performed appropriately and rigorously?

Reviewer #1: No

**Response: Thank you for your suggestions. For statistical analysis of the body weight, comparisons between time-based measurements within each group were performed with analysis of variance for repeated measurement ANOVA, and a P value <0.05 was considered to be statistically significant, as indicated in the section of 2.8 Statistical analysis and the revised Figure 1A.**

4. Have the authors made all data underlying the findings in their manuscript fully available?

The [PLOS Data policy](http://www.plosone.org/static/policies.action#sharing) requires authors to make all data underlying the findings described in their manuscript fully available without restriction, with rare exception (please refer to the Data Availability Statement in the manuscript PDF file). The data should be provided as part of the manuscript or its supporting information, or deposited to a public repository. For example, in addition to summary statistics, the data points behind means, medians and variance measures should be available. If there are restrictions on publicly sharing data—e.g. participant privacy or use of data from a third party—those must be specified.

Reviewer #1: Yes

5. Is the manuscript presented in an intelligible fashion and written in standard English?

PLOS ONE does not copyedit accepted manuscripts, so the language in submitted articles must be clear, correct, and unambiguous. Any typographical or grammatical errors should be corrected at revision, so please note any specific errors here.

Reviewer #1: Yes

6. Review Comments to the Author

Please use the space provided to explain your answers to the questions above. You may also include additional comments for the author, including concerns about dual publication, research ethics, or publication ethics. (Please upload your review as an attachment if it exceeds 20,000 characters)

Reviewer #1: All comments were addressed by the authors. For analyzing the the body weight, repeated measures should be done.

**Response: Thank you for your suggestions. For statistical analysis of the body weight, comparisons between time-based measurements within each group were performed with analysis of variance for repeated measurement ANOVA, and a P value <0.05 was considered to be statistically significant, as indicated in the section of 2.8 Statistical analysis and the revised Figure 1A.**

7. PLOS authors have the option to publish the peer review history of their article ([what does this mean?](https://journals.plos.org/plosone/s/editorial-and-peer-review-process#loc-peer-review-history)). If published, this will include your full peer review and any attached files.


If you choose “no”, your identity will remain anonymous but your review may still be made public.


Do you want your identity to be public for this peer review? For information about this choice, including consent withdrawal, please see our [Privacy Policy](https://www.plos.org/privacy-policy).

Reviewer #1: Yes: Hamideh Bashiri
